# Efficacy and safety of Xuefu Zhuyu Granules combined with western medicine in the treatment of angina pectoris of coronary heart disease: A study protocol of a randomized, double-blind, placebo-controlled clinical trial

**DOI:** 10.1097/MD.0000000000031235

**Published:** 2022-10-28

**Authors:** Dong Liu, Yunjie Zeng, Peng Liang, Yunlu Jiang, Su An, Pengcheng Ren

**Affiliations:** a The People’s Hospital of DaZu, Chongqing, China.

**Keywords:** angina pectoris, coronary heart disease, protocol, randomized controlled trial, Xuefu Zhuyu Granules

## Abstract

Despite advances in treatment strategies for coronary heart disease, angina pectoris remains a major cardiovascular disease causing death worldwide. For patients with angina pectoris of coronary heart disease, new or adjuvant treatment regimens are needed. The available evidence suggests that Xuefu Zhuyu Granules combined with Western medicine has advantages in the treatment of angina pectoris of coronary heart disease, but whether its efficacy has a placebo effect and whether it can be used as an adjuvant regimen for the treatment of angina pectoris of coronary heart disease remains controversial.

**Methods:**

This is a prospective, randomized, double-blind, placebo-controlled trial to study the efficacy and safety of Xuefu Zhuyu Granules combined with Western medicine in the treatment of angina pectoris of coronary heart disease. Participants will be randomly divided into a treatment group or a control group, and all patients will receive Western medicine treatment based on guideline recommendations. On this basis, the treatment group orally takes Xuefu Zhuyu Granules and the control group orally takes Xuefu Zhuyu Granules mimic, and are followed up for 24 weeks after 12 weeks of continuous treatment. The observation indexes include: cardiac function parameters (left ventricular end-diastolic diameter; left ventricular end-systolic diameter; left ventricular ejection fraction, blood lipid levels (total cholesterol; triacylglycerol; low-density lipoprotein cholesterol; high-density lipoprotein cholesterol), the number of angina attacks per week, total amount of nitroglycerin tablets taken, and adverse reactions. Finally, SPSS22.0 (IBM Company, New York, NY) software will be used for statistical analysis of the data.

**Discussion:**

This study will evaluate the efficacy and safety of Xuefu Zhuyu Granules combined with Western medicine in the treatment of angina pectoris of coronary heart disease. The results of this study will verify whether the efficacy of Xuefu Zhuyu Granules in the treatment of angina pectoris of coronary heart disease belongs to the placebo effect, which will also provide a reference for the clinical use of Xuefu Zhuyu Granules as a supplementary scheme for the treatment of angina pectoris of coronary heart disease.

## 1. Introduction

Coronary heart disease is one of the most common chronic diseases worldwide.^[1]^ According to statistics, 17.9 million people died of cardiovascular diseases worldwide in 2016, of which about 34% died of coronary heart disease.^[2,3]^ Angina pectoris is the most important and common type.^[4]^ It is a syndrome caused by acute myocardial ischemia and hypoxia.^[5]^ Studies have shown that angina pectoris of coronary heart disease is not treated in time, about 30% of patients will develop myocardial infarction, a serious threat to the life safety of patients.^[6]^ According to guidelines for the prevention and treatment of coronary heart disease, nitrates, β-blockers, calcium channel blockers, anticoagulants, and lipid-lowering drugs are standard and first-line therapeutic drugs for angina pectoris in coronary heart disease.^[7,8]^ Although these drugs rapidly improve clinical symptoms, there are some limitations in the face of the complex disease of coronary heart disease angina pectoris.^[9]^ And clinical studies have found that traditional anti-myocardial ischemia therapy does not reduce the incidence of cardiovascular adverse events, and 5% to 15% of refractory patients still have angina pectoris symptoms.^[9,10]^ Therefore, new treatment regimens are needed for the treatment of angina pectoris in coronary heart disease.

An important cause of angina pectoris in coronary heart disease is narrowing of small blood vessels supplying blood and oxygen to the heart,^[11]^ which is consistent with the Qi stagnation and blood stasis theories of traditional Chinese medicine.^[12]^ Therefore, regulating Qi and promoting blood circulation is the main therapeutic goal. Xuefu Zhuyu Granules is a traditional Chinese medicine prescription derived from the Qing Dynasty in China and has the effect of promoting Qi and activating blood circulation.^[13]^ Experimental studies have found that the potential mechanism of Xuefu Zhuyu Granules in the treatment of angina pectoris of coronary heart disease includes improving cardiac energy supply, reducing phospholipid peroxide, maintaining the polyunsaturated fatty acid metabolic peroxide balance and regulating amino acid metabolism, which has the effect of lowering blood lipids and whole blood viscosity.^[14]^ Xuefu Zhuyu Granules can greatly improve skin blood perfusion and improve the prognosis of patients with stable coronary heart disease.^[15]^ In China, the combination of traditional Chinese medicine and Western medicine is common, and such a treatment regimen can exert their respective advantages of traditional Chinese medicine and Western medicine to enhance the efficacy and reducing adverse reactions.^[16]^ The latest systematic review showed that Xuefu Zhuyu Decoction combined with Western medicine has advantages in the treatment of coronary heart disease, but its quality of evidence is low.^[17]^ Due to the lack of double-blind, placebo-controlled clinical studies, it remains controversial whether the efficacy of Xuefu Zhuyu Decoction is a placebo effect, and whether it has a synergistic effect and is safer in combination with Western medicine. This study will explore the efficacy and safety of Xuefu Zhuyu Granules combined with Western medicine for the treatment of angina pectoris of coronary heart disease through a prospective, randomized, double-blind, placebo-controlled trial.

## 2. Materials and Methods

### 2.1. Study design

**Figure 1.**
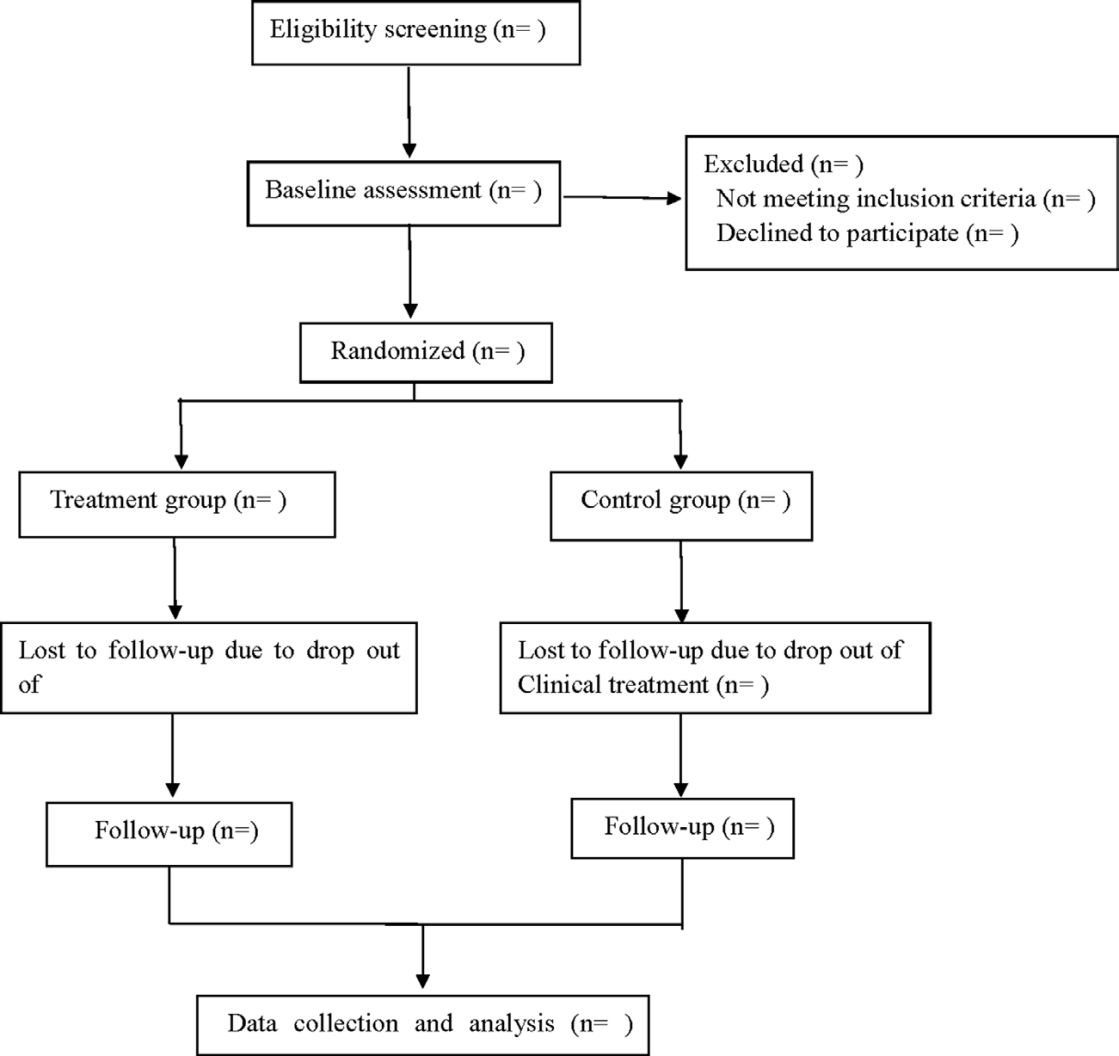
Flow diagram.

This is a prospective, randomized, double-blind, placebo-controlled study to explore the efficacy and safety of Xuefu Zhuyu Granules combined with Western medicine for the treatment of angina pectoris of coronary heart disease. Participants will be randomly divided into the treatment group and the control groups. Under the recommendation based on the guidelines, patients in both groups will receive a standard Western medicine treatment scheme. On this basis, the treatment group will receive Xuefu Zhuyu Granules orally, and the control group will receive Xuefu Zhuyu Granules mimic orally, follow up for 24 weeks after 12 weeks of continuous treatment. Flow diagram is shown in Figure 1, and study schedule is shown in Table 1. This study will follow the latest Consolidated Standards of Reporting Trials (CONSORT 2017)^[18]^ and Standard Protocol Items: Recommendations for Interventional Trials (SPIRIT checklist) 2013 statement.

**Table 1:** Study schedule.

Project	Stage				
	Screening period	Treatment period	Follow-up		
	Baseline	12-week	6-week	12-week	24-week
Record fill	√				
Fulfill inclusion criteria and exclusion criteria	√				
Sign informed consent	√				
Random allocation	√				
Treatment	√	√			
**Effectiveness observation**					
Cardiac function indexes	√	√	√	√	√
Blood lipid levels	√	√	√	√	√
Number of angina attacks per week	√	√	√	√	√
Total amount of nitroglycerin tablets	√	√	√	√	√
**Safety evaluation**					
Vital signs	√	√			√
Blood test and urinalysis	√	√			√
Liver and kidney function	√	√			√
Record of adverse event		√	√	√	√

### 2.2. Ethics and registration

The study protocol will be conducted in accordance with the Declaration of Helsinki and Ethical Guidelines for Clinical Research. This study has been reviewed by the Clinical Research Ethics Committee of our hospital and registered on the Thai Clinical Trials Registry (registration number: TCTR20220906002). Before randomization, all patients will be asked to sign an informed consent form, and they can choose whether to continue the trial at any time.

### 2.3. Sample size

The estimation of sample size is based on the mean and standard deviation of the scores of the left ventricular ejection fraction of patients after 12 weeks of treatment. Referring to the results of the preliminary experiment, 54.74 ± 11.17 in the treatment group and 48.58 ± 10.98 in the control group. Setting *α* = 0.05, using the different test, *β* = 0.20. It is calculated by PASS15.0 (NCSS Company, Kaysville, UT) software that 41 participants are needed in each group, with an estimated dropout rate of 20%, 52 patients will be included in each group.

### 2.4. Patients

#### 2.4.1. Diagnosis basis.

The diagnostic criteria for angina pectoris of coronary heart disease refer to the Nomenclature and Criteria for Diagnosis of Ischemic Heart Disease formulated by the World Health Organization.^[19]^ Traditional Chinese medicine (TCM) diagnostic criteria refer to the Diagnostic and Therapeutic Criteria for TCM diseases.^[20]^

#### 2.4.2. Inclusion criteria.

1. Meet the diagnostic criteria of coronary heart disease angina pectoris in traditional Chinese medicine and Western medicine, coronary angiography or coronary angiography showed at least one coronary artery stenosis and catheter stenosis ≥50%.
2. Aged ≥18 years old, and ≤70 years old.
3. Canadian Cardiovascular Society classification of angina pectoris is grade I–III.
4. Angina pectoris attack frequency ≥2 times/week, and ≤6 times/day.
5. Patients agreed to join this study, and signed informed consent.

#### 2.4.3. Exclusion criteria.

1. Patients who have undergone coronary artery bypass surgery.
2. Patients with other serious diseases (such as severe cardiopulmonary dysfunction, tumor, etc.) or alanine transaminase, aspartate aminotransferase or Cr reaching 1.5 times of the normal upper limit.^[21]^
3. Patients with other diseases that may increase the risk of bleeding (such as cerebral hemorrhage, upper gastrointestinal bleeding), the platelet count decreased, coagulation dysfunction, etc.
4. Pregnant, preparing for pregnancy or lactating women.
5. Patients are allergic to the use of drugs in the study.
6. Participated in or are participating in other clinical trials in the past 1 month.

### 2.5. Study design

Participants will be divided into a treatment group or control group in a 1:1 ratio by a central network-based randomization tool in this study. Independent statisticians will generate random sequences using SAS 9.3 software (SAS Institute, Cary, NC). When the research assistant enters the patient information on the tablet computer, he/she will be assigned a random number and complete the randomization according to the result of the assignment. Before the end of the study, all persons will be blinded to the group assignment, including the researchers and the patient.

### 2.6. Intervention measures

According to the 2014 American College of Cardiology/American Heart Association Guidelines for the Diagnosis and Treatment of Non-ST-Segment Acute Coronary Syndrome, all patients will receive dual antiplatelet therapy (aspirin 100 mg/days + clopidogrel 75 mg/days) and unfractionated heparin, and statins, angiotensin-converting enzyme inhibitors, β-blockers, and nitrates according to the guidelines.^[22]^ All these basic treatments will be recorded in detail in the patient’s medical records and case report forms (CRFs).

The treatment group will orally take Xuefu Zhuyu Granules (Chenpai Pharmaceutical, Jiangsu, China), 1 bag/time, 3 times/day. The control group will orally take Xuefu Zhuyu Granules mimic (Xingreen Pharmaceutical, Sichuan, China, its appearance and taste are the same as Xuefu Zhuyu Granules), 1 bag/time, 3 times/day. Patients in both groups will receive the standard of treatment for 12 weeks.

### 2.7. Outcomes

1. Cardiac function indexes: left ventricular end-diastolic diameter, left ventricular end-systolic diameter and left ventricular ejection fraction will be measured by color doppler ultrasonic diagnostic apparatus (GE-VIVID7, US).
2. Blood lipid levels: Fasting veins of the patients will be collected in the morning before and after treatment and during follow-up, and blood lipid index, including total cholesterol; triacylglycerol; low-density lipoprotein cholesterol and high-density lipoprotein cholesterol, were measured by an automatic biochemical analyzer (HITEC-7100, Japan).
3. Number of angina attacks per week, total amount of nitroglycerin tablets taken during the study.

We will collect the above outcome measures at baseline, at the end of treatment, and at the 6th, 12th and 24th week of follow-up.

### 2.8. Safety evaluation

Safety evaluation includes vital signs, blood, urine, and stool routine tests, liver and kidney function, bleeding spots, skin ecchymosis, and adverse events. Any discomfort experienced by the patient during the study will be reported to the investigator, and details of all adverse events will be recorded in the CRF. Safety assessors will assess the safety of both options at the end of the study.

### 2.9. Data management and quality control

Trained researchers will collect research data throughout the study and record in the CRF. Any amendment to the study protocol will be applied in advance and approved by the Ethics Committee of our hospital. To ensure the reliability and confidentiality of the study data. All data will be stored in an independent storage room. Access to the database will be restricted to researchers on this study team. Participants’ information will not be made publicly available and shared without written permission from participants.

### 2.10. Statistical analysis

Outcome measure data will be statistically analyzed by the full analysis set and per-protocol set. Safety evaluation data will be based on safety set. Statistical evaluation of full analysis set will follow intent-to-treat. Data from this study will be statistically analyzed using SPSS22.0 (IBM Company, New York, NY) software. Count and grade data will be expressed as percentage (%), 2-test or non-parametric test; measurement data will be expressed as mean ± standard deviation or interquartile range M (P25, P75), normal distribution will be analyzed by *t*-test, and skewed distribution will be analyzed by non-parametric test; If the data at multiple time points are normal and have homogeneous variance, the analysis of variance will be used, otherwise the generalized estimating equation will be used; The difference will be considered statistically significant when *P <* .05.

## 3. Discussion

Acute myocardial ischemia and hypoxia caused by insufficient coronary artery blood supply due to increased cardiac load or coronary artery spasm are the pathological factors of angina pectoris.^[15,23]^ Therefore, promoting Qi, activating blood circulation, and removing blood stasis is the principles of TCM in the treatment of angina pectoris of coronary heart disease. Xuefu Zhuyu Granules is composed of 11 kinds of Chinese herbal medicines, including Tao Ren (*Semen Persicae*),Hong Hua (*Flos Carthami*), Dang Gui(*Radix Angelicae Sinensis*), Di Huang (sheng) (*Radix Rehmanniae*), Niu Xi (*Radix Acanthopanacis Bidentatae*), Chuan Xiong (*Rhizoma Chuanxiong*), Jie Geng (*Radix Platycodonis*), Chi Shao (*Radix Paeoniae Rubr*a), Zhi qiao (*Fructus Aurantii*), Gan Cao (*Radix Glycyrrhizae*), and Chai Hu (*Radix Bupleuri*), it has the functions of promoting blood circulation,removing blood stasis, promoting Qi and relieving pain.^[24]^ Xuefu Zhuyu Granules can lower blood lipid levels, slow down the inflammatory reaction, improve hemorheology, reduce plaques, and resist atherosclerosis.^[25]^ Although current evidence suggests Xuefu Zhuyu Granules is beneficial in patients with angina pectoris of coronary heart disease.^[17]^ However, the application of Xuefu Zhuyu Granuless in angina pectoris of coronary heart disease is limited by the quality of the study (such as incorrect randomization method, no blind method, etc.). In previous studies, the scale score is usually used to evaluate the cardiac function indexes of patients, which are subjective.^[26]^ Therefore, this study intends to explore the efficacy and safety of Xuefu Zhuyu Granules combined with Western medicine in the treatment of angina pectoris of coronary heart disease through a standard randomized controlled trial, and objectively evaluate the impact of this regimen on cardiac function through imaging indicators.

This study also has some shortcomings: although our follow-up time is 24 weeks, which is much longer than other clinical studies on the same theme, the effect of Xuefu Zhuyu Granules combined with Western medicine on major cardiovascular adverse events may still not be observed; In addition, this is a single-center study, and the results may have regional differences.

## Author contributions

**Conceptualization:** Dong Liu and Yunjie Zeng

**Data curation:** Peng Liang and Yunlu Jiang

**Formal analysis:** Su An and Pengcheng Ren

**Funding acquisition:** Pengcheng Ren

**Software:** Dong Liu and Yunjie Zeng

**Supervision:** Su An and Pengcheng Ren

**Writing - original draft:** Dong Liu and Yunjie Zeng

**Writing - review & editing:** Peng Liang and Yunlu Jiang
